# CD24: A Novel Target for Cancer Immunotherapy

**DOI:** 10.3390/jpm12081235

**Published:** 2022-07-28

**Authors:** Emmanouil Panagiotou, Nikolaos K. Syrigos, Andriani Charpidou, Elias Kotteas, Ioannis A. Vathiotis

**Affiliations:** Section of Medical Oncology, Third Department of Internal Medicine, National and Kapodistrian University of Athens, 11527 Athens, Greece; em_panagiotou@outlook.com (E.P.); nsyrigos@hsph.harvard.edu (N.K.S.); dcharpidou@yahoo.gr (A.C.); ilkotteas@med.uoa.gr (E.K.)

**Keywords:** CD24, siglec-10, immunotherapy

## Abstract

Cluster of differentiation 24 (CD24) is a small, highly glycosylated cell adhesion protein that is normally expressed by immune as well as epithelial, neural, and muscle cells. Tumor CD24 expression has been linked with alterations in several oncogenic signaling pathways. In addition, the CD24/Siglec-10 interaction has been implicated in tumor immune evasion, inhibiting macrophage-mediated phagocytosis as well as natural killer (NK) cell cytotoxicity. CD24 blockade has shown promising results in preclinical studies. Although there are limited data on efficacy, monoclonal antibodies against CD24 have demonstrated clinical safety and tolerability in two clinical trials. Other treatment modalities evaluated in the preclinical setting include antibody–drug conjugates and chimeric antigen receptor (CAR) T cell therapy. In this review, we summarize current evidence and future perspectives on CD24 as a potential target for cancer immunotherapy.

## 1. Introduction

Checkpoint immunotherapy with programmed cell death protein 1 (PD-1)/programmed death-ligand 1 (PD-L1) and/or cytotoxic T-lymphocyte-associated protein 4 (CTLA-4) inhibitors has altered the treatment landscape in medical oncology, thus improving patient outcomes [1,2]. Additional immune checkpoint molecules that facilitate tumor immune evasion represent potential targets for novel immunotherapy strategies [3].

Cluster of differentiation 24 (CD24), also known as heat-stable antigen (HSA), is a highly glycosylated 31–34 amino acid protein attached to the cell surface by a glycosylphosphatidylinositol anchor [4]. It is normally expressed on B and T lymphocytes, monocytes, and granulocytes [4,5] as well as epithelial, neural, and muscle cells [5]. CD24 represents a costimulatory molecule for CD4+ T cell proliferation [6]. It has been shown to have a proapoptotic role in B cell precursors as well as in differentiated B cells, possibly through the activation of the MAPK pathway [7,8]; CD24 is a marker for B10 regulatory B cells, which restrain the immune response by regulating T cell activity [9]. In addition, CD24 is highly expressed in regulatory T cells and may regulate their immunosuppressive activity [10]. It is also involved in the differentiation of CD8+ T cells [11]. Thus, decreased CD24 expression may lead to uncontrolled T cell proliferation, with implications in autoimmunity [12]. Furthermore, CD24 interaction with P-selectin [13] mediates leukocyte binding to endothelial cells and activated platelets [14], therefore promoting cell migration [15]. CD24 also regulates the activity of several other adhesion molecules, including integrins, by affecting their localization in the cell membrane, possibly by activating Src-related kinases [15,16]. While it is also involved in the differentiation of neural stem cells [17], CD24 is transiently expressed during the development of the human central nervous system [18,19]. As a matter of fact, the activity of L1CAM (CD171), a neural signal transduction molecule which regulates neurite outgrowth, appears to be dependent upon its interaction with CD24-associated sialic acids [20].

CD24 also plays an important role in tumorigenesis. Surface CD24 expression in tumor cells has been linked with alterations in multiple oncogenic signaling pathways, including Src/STAT3 [21], EGFR [22], HER2 [23], Ras-like GTPase [24], MAPK [25,26], AKT/mTOR [27], WNT/β-catenin [28], and miRNA-related pathways [25]. Moreover, the cytoplasmic accumulation of CD24 may be involved in tumor cell proliferation, including p53 inactivation [4]. In this regard, the overexpression of CD24 has been documented in several malignancies, including breast [29,30], lung [31], colorectal [32], hepatocellular [27], pancreatic [33], ovarian [30], urothelial [34], prostate [35], and head and neck cancer [36], as well as in primary central nervous system (CNS) tumors [37,38] and hematologic malignancies [39,40,41] (Table 1).

Notably, the expression of CD24 in solid tumors has been correlated with worse prognosis, including the presence of more aggressive features and their metastatic spread in vivo [44]; in fact, CD24 expression has been shown to promote tumor growth and malignant behavior in a dose-dependent manner [45]. Tumor cell CD24 interaction with P-selectin on endothelial cells may account for the latter [4,46]. Additional mechanisms that depend upon the expression of CD24 and are implicated in tumor cell extravasation and metastasis include rolling on E-selectin [47] and binding to fibronectin in the extracellular matrix [47]. CD24 represents a target of the transcription factor hypoxia inducible factor-1α (HIF-1α), a molecule commonly upregulated in hypoxic conditions, including during primary tumor growth and metastasis [48]. Intriguingly, metastatic tumor growth has been shown to be inhibited when CD24 activity is suppressed [34]. Finally, the surface expression of CD24 in peripheral blood leukocytes has been shown to be both sensitive and specific for the detection of colorectal adenoma and cancer in individuals undergoing colonoscopy [49]. Combined with peripheral blood CD11b expression, surface CD24 has also been evaluated as a potential screening tool for the detection of hematologic malignancies [50]. 

Its multifaceted role in both the regulation of the immune response and tumorigenesis has made CD24 a compelling target for the immunotherapy of cancer. In this review, we sought to summarize the available data on the role of CD24 in chemoresistance and antitumor immunity. In addition, we present clinical trials that have assessed the efficacy of CD24 blockade in cancer, either alone or in combination with other treatment modalities.

## 2. Resistance to Chemotherapy

In the preclinical setting, the overexpression of CD24 has been associated with resistance to several chemotherapy agents. Leukemia stem cells with CD24 surface expression appear to be resistant to doxorubicin, possibly through the activation of the Wnt/β-catenin and PI3K/Akt pathways [51]. Likewise, downstream activation of the PI3K/Akt pathway by CD24 has been shown to reduce the sensitivity of retinoblastoma cells to vincristine [52]. Thus, the inhibition of autophagy through the PI3K/Akt pathway seems to be involved in the development of CD24-mediated chemoresistance [53]. Another mechanism of resistance may involve the activation of MAPK-related pathways, resulting in temporary inhibition of cell proliferation [54]. CD24 may also enhance cytotoxicity from DNA-damaging agents through the disruption of NF-κB signaling [55]. CD24+ head and neck squamous cell carcinoma (HNSCC) cell lines were resistant to cisplatin, and this effect was reversed by CD24 inhibition [56]. Additional preclinical data support chemoresistance to doxorubicin and cisplatin for CD24+ ovarian cancer [57] and to gemcitabine for CD24+ pancreatic cancer cell lines [58]. Moreover, the overexpression of CD24 has been linked with worse outcomes following chemoradiotherapy in patients with glioblastoma [59] and locally advanced HNSCC [36]. 

CD24-induced chemoresistance may be tissue-dependent. The induced expression of CD24 in endometrial cell lines increased resistance to doxorubicin and paclitaxel [60]. Furthermore, treatment with anti-CD24 antibodies conferred increased sensitivity to multiple chemotherapy agents in colorectal cancer models. However, this is not the case for early breast cancer, where the increased expression of CD24 has demonstrated conflicting results across studies [61,62]. The latter may be due to polymorphisms in the CD24 gene, which may be culpable of discordances in tumor immune infiltration and thus explain the differences in the pathologic response to neoadjuvant chemotherapy [63].

Finally, the overexpression of CD24 has been linked with resistance to targeted therapies. In melanoma, the increased expression of CD24 was associated with resistance to BRAF inhibitors [64]. In addition, CD24 was shown to promote resistance to sorafenib in hepatocellular carcinoma [27]. CD24 was also shown be upregulated and to promote resistance to EGFR inhibition in lung adenocarcinoma cells resistant to gefitinib [65,66]. In addition, CD24 polymorphisms were associated with differences in survival outcomes after neoadjuvant treatment, which included bevacizumab in resected metastatic colorectal cancer [67].

## 3. Implications in Antitumor Immunity

Siglecs, or sialic-acid-binding immunoglobulin-like lectins, are a family of surface receptors that are expressed by immune cells and bind sialic acid-containing glycans, also known as sialoglycans [68]. Siglecs have been implicated in various components of the immune response, such as in the regulation of immune cell proliferation, survival, T cell and eosinophil activity, antigen presentation, and endocytosis signaling [69]. CD24 is the main ligand for Siglec-10 [30]; Siglec-10 is widely expressed by B cells, activated T cells, and monocytes [28]. The CD24/Siglec-10 complex engages danger-associated molecular pattern (DAMP) molecules, released as a result of tissue injury, in order to initiate a signaling cascade that is mediated by SHP-1 and/or SHP-2 phosphatases associated with the two immunoreceptor tyrosine-based inhibition motifs (ITIMs) in the cytoplasmic tail of Siglec-10. This inhibits TLR-mediated inflammation, which ultimately leads to the suppression of the immune response following tissue damage [70,71]. Selective interaction with DAMPs but not with pathogen-associated molecular pattern molecules (PAMPs), which are related to the recognition of “non-self” patterns associated with infection, provides the biologic basis for a differential response between healthy and neoplastic tissue as a result of CD24 blockade [71,72]. As a matter of fact, inactivating CD24 alterations may confer protection against autoimmune diseases such as systemic lupus erythematosus and multiple sclerosis [73]. The anti-inflammatory effects of CD24 activation have also been explored as a strategy to counteract immune-related adverse events (NCT04552704, NCT04060407), graft-versus-host disease [74], and as of late, COVID-19 [75]. The effects of CD24 signaling on immune cell activity are summarized in Table 2.

Several malignancies have been found to overexpress sialyated glycans, which connect with Siglec-10 and employ the CD24/Siglec-10 interaction as a means of tumor immune evasion [76]. This mimics the innate “recognition of self” signal that is emitted by injured tissues to inhibit inflammation and allow for tissue healing [76]. Aberrant sialyation has been linked with uncontrolled cell proliferation and malignant potential, as well as tumor immune evasion [77]. Mouse sepsis models have provided a possible mechanism for the inhibition of the CD24/Siglec-10 interaction and the resulting immunosuppression through the disruption of CD24 sialyation with the use of recombinant bacterial sialidases; however, this strategy harbors the risk of increasing susceptibility to sepsis secondary to bacterial infection [78].

In addition, the surface expression of CD24 has been described as a “do not eat me” signal, inhibiting phagocytosis by infiltrating Siglec-10-expressing macrophages [30]. In contrast, the loss of CD24 expression has been shown to increase phagocytosis by macrophages expressing Siglec-10 in renal clear cell carcinoma [79]. Furthermore, a decrease in Siglec binding to surface sialic acids promotes macrophage-mediated phagocytosis [80]. Silencing NPM/B23, a protein that induces CD24 expression, promotes macrophage-mediated phagocytosis by downregulating CD24, while the restoration of CD24 expression inhibits phagocytosis and therefore promotes immune evasion [81].

Natural killer (NK) cells have been shown to selectively eradicate less differentiated cells such as CD24+ ovarian cancer cells [57] and cancer stem cells [82]. This antitumor effect is largely dependent on the activation of the Natural Killer Group 2D (NKG2D) receptor [82]. NKG2D-mediated NK cell cytotoxicity has been correlated with intracellular reactive oxygen species (ROS) formation, with cells that have low ROS levels being more sensitive to NK cell-mediated killing [83]. The CD24/Siglec-10 interaction has been linked with NK cell dysfunction in hepatocellular carcinoma and could provide another pathway for CD24-mediated immune evasion [84]. 

The correlation between CD24 and PD-1/PD-L1 immune checkpoints has not been clearly established at this point. The overexpression of PD-L1 seems to downregulate surface CD24 in breast cancer cells [85]. Moreover, high PD-1 expression was correlated with decreased CD24 expression in B cells infiltrating hepatocellular carcinoma tumors, which resulted in decreased cytokine production [86]. Furthermore, treatment with CD24Fc reduced infiltration by PD-1-expressing T cells in vivo [87]. However, this was not the case in patients with NSCLC [88]. Although early indications for the interaction between CD24 and PD-(L)1 point towards tumor type specificity, more data are needed to draw definitive conclusions.

CD44 is a cell surface glycoprotein that acts as an adhesion molecule by binding to hyaluronic acid [89]. It is normally expressed on all hematopoietic as well as most epithelial cells [90]. While its possible role in tumorigenesis is currently under investigation, CD44 is widely used as a marker of cancer stem cells [91]. By regulating P- and L-selectin activity, CD44 is thought to be involved in hematogenous dissemination, including leukocyte recruitment and metastatic tumor spread [92]. More specifically, it appears to control tumor infiltration by the intratumoral movement of cytotoxic T cells [93] and may exert an immune suppressive effect upon its interaction with osteopontin [94,95]. Intratumoral movement is also regulated by the CD44/hyaluronic acid interaction, which is involved in the reorganization of the cytoskeleton that is necessary for cell migration [89]. CD44+/CD24–/low cells have been described as breast [96] and prostate cancer [97] stem cells; stem-like properties, including increased proliferation [96], angiogenesis [98], and resistance to chemotherapy [99], have been attributed to CD44+/CD24–/low NSCLC [100], and ovarian cancer cells as well [101]. However, the loss of CD24 expression does not appear to be necessary for cancer stemness. As a matter of fact, CD44+/CD24+ pancreatic cancer cells that express epithelial-specific antigens appear to possess several stem-like properties that have led them to be considered as tumor-initiating cells [102]. Targeting those cells in particular resulted in significant antitumor activity in vivo and could evolve into a potential strategy for pancreatic cancer immunotherapy [103]. Furthermore, CD44+/CD24+ melanoma cells have been shown to have greater tumor-forming potential in vivo than CD44+/CD24–, suggesting that the increased malignant potential might be attributable to CD24 expression [104].

## 4. CD24 Inhibition in Solid Tumors

### 4.1. Preclinical Data

Treatment strategies that have employed the inhibition of the CD24 immune checkpoint have been evaluated in the preclinical setting. In vivo studies are summarized in Table 3.

#### 4.1.1. Monoclonal Antibodies

Monoclonal antibodies targeting CD24 have been used either with or without chemotherapy in various solid tumor models. ALB9 prevented lung metastases from CD24-overexpressing human urothelial cancer cells, with lung colonization restarting promptly after treatment discontinuation [34]. SWA11, which is another monoclonal antibody against CD24, reduced proliferation in human lung [21,105], ovarian [21,105], and pancreatic cancer cell lines [21] and impeded tumor growth in human colorectal cancer xenograft models [106]. Monotherapy with SWA11 has been shown to decrease tumor volume in several human xenograft models [21]. Moreover, pretreatment with SWA11 increased the antitumor efficacy of gemcitabine in vivo, particularly by promoting macrophage infiltration and disrupting angiogenesis [105]. SWA11 has been shown to increase the antitumor efficacy of multiple chemotherapy agents, including oxaliplatin, 5-fluorouracil, irinotecan, paclitaxel, and doxorubicin [106]. Dual CD24 inhibition with SWA11 and ML-5 suppressed the proliferation of CD24+ pancreatic cancer cell lines, with the antiproliferative effect correlating with CD24 expression levels [33]. Clone SN3 promoted phagocytosis in ovarian and breast cancer patient-derived CD24+ cell lines, and it increased survival in vivo through the macrophage-mediated inhibition of tumor growth [30]. 

The G7 monoclonal antibody designed against CD24 has demonstrated some antitumor activity as well [107,113]. Its combination with cetuximab exhibited enhanced antitumor efficacy, possibly through disruption of STAT3 signaling [107]. Concomitant manipulation of CD24 and CD47 has also been investigated as a potential treatment for glioblastoma in the preclinical setting [117]. The development of a bispecific antibody against the NK receptor ligand MICA and G7 has also shown promising preliminary results [108,118]. This approach employed the increased expression of CD24 in hepatocellular carcinoma tumor cells in order to recruit NK cells at the tumor site [108,118] and promote NK cell-mediated cytotoxicity, consequently showing substantial antitumor activity in vivo.

Recombinant antibodies targeting CD24 have also been tested. Single-chain variable fragments (scFvs), selected from a total RNA library from lymphocytes of breast cancer patients, showed high selectivity for CD24 and CD44 and produced a synergistic effect when administered in combination with epirubicin [109]. ScFvs have received FDA approval in malignancies, including blinatumomab for acute lymphoblastic leukemia [119], and represent an alternative method for targeting CD24.

#### 4.1.2. Antibody–Drug Conjugates

Antibody–drug conjugates have also shown promising results in preclinical studies. As such, the conjugate of an anti-CD24 monoclonal antibody with deglycosylated ricin A-chain delayed tumor growth in a small cell lung cancer xenograft model [110]. A second antibody–deglycosylated ricin A-chain conjugate produced complete remissions that were durable and increased survival in a Burkitt’s lymphoma mouse model [111]. Anti-CD24 monoclonal antibodies have also been conjugated with Pseudomonas-derived exotoxin; this approach produced dose-dependent cytotoxicity in CD24-expressing colorectal cancer cells and inhibited tumor growth in xenograft models, with the results replicated in a second preclinical study [45,112].

A humanized murine anti-CD24 monoclonal antibody conjugated with monomethyl auristatin E (MMAE), a tubulin inhibitor, exhibited antitumor efficacy in mouse hepatocellular carcinoma models [113]. MMAE represents a validated conjugate candidate, that has demonstrated activity in hematologic malignancies and has gained regulatory approval by the FDA [120]. A conjugate bearing doxorubicin induced cell cycle arrest in CD24+ hepatocellular carcinoma cells and impeded tumor growth in mice [114]. Finally, an antibody–nitric oxide conjugate delayed hepatocellular carcinoma tumor growth in mice by nitrating the mitochondrial protein Cyt c and promoting apoptosis [115]. More importantly, the conjugated form exhibited superior antitumor activity as compared with the unconjugated anti-CD24 monoclonal antibody itself [115]. 

#### 4.1.3. Chimeric Antigen Receptor (CAR) T Cell Therapy

Following the successful implementation of CAR T cell immunotherapy in hematologic malignancies [121], some preclinical studies have employed engineered immune cells to target CD24 in solid tumors. Anti-CD24 CAR T cell therapy was effective in reducing tumor growth and metastasis in human pancreatic adenocarcinoma xenografts in mice [116]. The treatment was effective even in CD24 subclone-bearing tumors [116], suggesting that targeting pancreatic cancer stem cells with the use of this approach could be a viable treatment strategy for the therapy of pancreatic cancer.

Beyond T cells, NK cells have also demonstrated cytotoxic activity against ovarian cancer cell lines and patient-derived ovarian cancer cells [122]. Another strategy was the use of dendritic cells loaded with antibody-coated cancer cells, which targets different surface antigens, including CD24, to cross-present tumor antigens to CD8+ T cells [123]. This approach promoted T cell-mediated cytotoxicity in melanoma and ovarian cancer cell lines.

### 4.2. Clinical Trials

To this day, two clinical trials evaluating CD24-inhibiting agents have been completed in patients with cancer. The first one was a single-arm phase 1/2 study that enrolled 58 patients with severe B cell lymphoproliferative disease after bone marrow or organ transplantation [124,125]. The patients received a combination of two monoclonal antibodies: ALB9 targeting CD24 and BL13 targeting CD21 [124,125]. Overall, the study treatment was well-tolerated, with the most common adverse events being grade ≥ 3 transient neutropenia (42%) and grade 2 fever (22%) during the first infusion [124,125]. Grade 3 sepsis, diarrhea, vomiting, and thrombocytopenia were each reported in one patient. Combination therapy showed substantial clinical activity, with complete remission achieved in 61% of the cases [125]. After a median follow-up of 61 months, the overall survival rate was 46% [125]. However, the study treatment showed limited activity in the central nervous system [124,125]. As a result, this strategy has not been explored any further, and current treatment options for B cell lymphoproliferative disease arising post transplantation include the reduction of immunosuppression, rituximab, chemotherapy, or their combination [126]. 

The second study was a single-arm, single-institution phase 1/2 study involving 36 patients with primary hepatocellular carcinoma that had been subjected to surgical resection [127]. Study participants received adjuvant therapy with autologous transfusions of dendritic cells and cytokine-induced T cells loaded with the CD24 peptide [127]. The treatment was safe, with the most common adverse event being transient fever (<grade 3) in 19% of the patients [127]. No grade 3 or higher adverse events were reported [127]. The 4-year overall survival rate was 47% and 53% in patients receiving study treatment two and four times, respectively [127]. 

Additional clinical trials evaluating agents that target the CD24 immune checkpoint in patients with melanoma or advanced solid tumors have been registered (NCT04552704, NCT04060407). Τhese trials used a recombinant fusion protein, CD24Fc, which activates the CD24/Siglec-10 pathway [128], in combination with immunotherapy; their aim was to evaluate whether combination therapy would reduce the incidence and/or severity of immune-related adverse events. One of these trials was terminated early (NCT04552704), while the other was withdrawn before recruitment started (NCT04060407). Clinical trials of CD24-targeting agents in malignancies are summarized in Table 4.

## 5. Conclusions

CD24 represents a promising target for cancer immunotherapy. The overexpression of CD24 has been documented in several tumor types and the activation of the CD24/Siglec-10 axis has been shown to promote tumor immune evasion through the suppression of the cytotoxic T cell function and macrophage-mediated phagocytosis. Preclinical efforts to inhibit CD24 signaling have employed monoclonal antibodies, antibody–drug conjugates, and CAR T cell therapy. Although clinical evidence regarding the efficacy of CD24 blockade is currently limited, the compelling preclinical data in various tumor types—including but not limited to those with dismal prognosis and limited efficacy of PD-1/PD-L1 axis inhibition such as in pancreatic and ovarian cancer—as well as the pressing clinical need for novel treatment strategies might provide enough justification for the accelerated clinical testing of CD24 axis inhibitors. A potential caveat to that would be the lack of established biomarkers for the prediction of response to treatment with anti-CD24 antibodies. There is currently no standardized method for determining CD24 positivity, with some studies using flow cytometry and others employing immunohistochemical expression, with substantial heterogeneity. Finally, more preclinical data are required to delineate a potential role for a concomitant CD24 and PD-1/PD-L1 axis blockade.

## Figures and Tables

**Table 1:** Association between CD24 overexpression and disease features across different tumor types.

Tumor Type	CD24 Overexpression	Disease Characteristics	Outcome	Reference
Hepatocellular carcinoma	High IHC expression	NA	Decreased OS	[27]
Breast carcinoma	Moderate/high-intensity IHC staining or present in >26% of cells	Luminal A subtype	Decreased OS	[29]
Breast carcinoma	mRNA expression >median	TNBC subtype	Decreased OS	[30]
Ovarian carcinoma	mRNA expression >median	NA	Decreased RFS	[30]
Colorectal adenocarcinoma	Moderate/high IHC staining, mRNA expression >90th percentile	NA	Increased OS	[42]
Urothelial carcinoma	Moderate/high-intensity IHC staining in >10% of cells	High grade, stage	NA	[43]
Prostate adenocarcinoma	Any IHC staining	High stage	Decreased PSA relapse time	[35]
Oral squamous cell carcinoma	IHC staining in >10% of cells	NA	Decreased ORR to neoadjuvant therapy	[36]
Multiple myeloma	BM PC expression >5% by flow cytometry	NA	Increased PFS, OS	[41]

Abbreviations—BM: bone marrow, CRC: colorectal carcinoma, HCC: hepatocellular carcinoma, IHC: immunohistochemistry, ORR: objective response rate, OS: overall survival, PC: plasma cell, PFS: progression-free survival, RFS: relapse-free survival.

**Table 2:** Effects of CD24 signaling different subtypes of immune cells.

Immune Cell Subype	Effect	Proposed Mechanism	Outcome
T cells	Regulation of proliferation	Inhibition of rapid T cell proliferation in lymphopenic hosts	Inhibition
T cells	Downregulation of Th1, upregulation of Treg cells	CD24+ Breg cells	Inhibition
T cells	Promotion of the differentiation of memory/effector T cells	Costimulatory signal for naive CD8+ T cells	Activation
NK cells	Reduced NK cell cytotoxicity	Siglec-10-mediated	Inhibition
Macrophages	Inhibition of phagocytosis	Siglec-10-mediated	Inhibition
Monocytes/neutrophils	Hematogenous spread	P-selectin-mediated cell adhesion to activated endothelial cells or platelets	Activation
Dendritic cells	Suppression of immune response to tissue damage	Inhibition of TLR-mediated inflammation via Siglec-10 interaction	Inhibition

**Table 3:** Preclinical studies in vivo with agents that target CD24.

Tumor Type	Anti-CD24 Agent	Other Agents	Result	Proposed Mechanism	Reference
Urothelial carcinoma	ALB9	NA	Tumor growth inhibition	Inhibition of P-selectin-mediated metastatic dissemination	[34]
Lung adenocarcinoma	SWA11	NA	Tumor growth inhibition	Inhibition of Src/STAT3 signaling	[21]
Pancreatic adenocarcinoma	SWA11	NA	Tumor growth inhibition	Inhibition of Src/STAT3 signaling	
Ovarian carcinoma	SWA11	NA	Tumor growth inhibition	Inhibition of Src/STAT3 signaling	
Lung adenocarcinoma	SWA11	Gemcitabine	Tumor growth inhibition, increased efficacy of gemcitabine	Modification of intratumoral cytokine microenvironment	[105]
Ovarian carcinoma	SWA11	NA	Tumor growth inhibition	Modification of intratumoral cytokine microenvironment	
Colorectal adenocarcinoma	SWA11	Paclitaxel, doxorubicin, 5-fluorouracil, oxaliplatin, irinotecan	Tumor growth inhibition, increased efficacy of chemotherapeutic agents	Inhibition of Ras pathway	[106]
Breast carcinoma	SN3	NA	Tumor growth inhibition	Promotion of phagocytosis by Siglec-10-expressing macrophages	[30]
Lung adenocarcinoma	G7mAb	Cetuximab	Improved survival	Inhibition of STAT3 signaling by dual targeting of CD24 and EGFR	[107]
Hepatocellular carcinoma	G7mAb	Cetuximab	Tumor growth inhibition, improved survival	Inhibition of STAT3 signaling by dual targeting of CD24 and EGFR	
Colorectal adenocarcinoma	G7mAb	Cetuximab	Tumor growth inhibition, improved survival	Inhibition of STAT3 signaling by dual targeting of CD24 and EGFR	
Hepatocellular carcinoma	rG7S-MICA	NA	Tumor growth inhibition	NK cell recruitment through MICA/NKG2D pathway	[108]
Breast carcinoma	scFvs	Epirubicin	Tumor growth inhibition, increased efficacy of epirubicin	Targeting of CD44+/CD24+ cells	[109]
Small cell lung cancer	SWA11-SPDB-dg.ricin A chain	NA	Tumor growth inhibition	Targeted, ricin-mediated toxicity	[110]
Burkitt’s lymphoma	SWA11.dgA	NA	Durable complete remissions	Targeted, ricin-mediated toxicity	[111]
Colorectal adenocarcinoma	SWA11-ZZ-PE38	NA	Tumor growth inhibition	Targeted, exotoxin-mediated cytotoxicity	[112]
Hepatocellular carcinoma	hG7-BM3-VcMM	NA	Tumor growth inhibition	Targeted, MMAE-mediated cytotoxicity	[113]
Hepatocellular carcinoma	G7mAb-DOX	NA	Tumor growth inhibition, improved survival	Targeted, doxorubicin-mediated cytotoxicity	[114]
Hepatocellular carcinoma	HN-01	NA	Tumor growth inhibition, improved survival	Targeted, intracellular release of nitric oxide	[115]
Pancreatic adenocarcinoma	CAR-redirected anti-CD24 T-cells	NA	Tumor growth inhibition, improved survival	T-cell mediated cytotoxicity	[116]

**Table 4:** Clinical trials with agents targeting CD24 in patients with cancer.

Trial Identifier	Inclusion	Agent	Phase	Setting	Primary Outcome	Enrollment	Status	Results
NCT04552704	Advanced Solid Tumors	CD24 agonist	I/II	Any	Safety, tolerability, recovery from irAEs	78	Active, not recruiting	No
NCT04060407	Unresectable or metastatic melanoma	CD24 agonist, nivolumab, ipilimumab	Ib/II	Any	Safety, tolerability	0	Withdrawn	No
NA	Posttransplant BLPD	ALB9, BL13	I/II	First line	Safety, tolerability	58	Completed	Yes
NA	Resected HCC	CD24-loaded DC/CIK autotransfusion	I/II	Adjuvant	Safety, efficacy	36	Completed	Yes

## Data Availability

Not applicable.
